# Research status of visuospatial dysfunction and spatial navigation

**DOI:** 10.3389/fnagi.2025.1609620

**Published:** 2025-05-14

**Authors:** Rui Bao, Shijie Chang, Ruixiang Liu, Yunning Wang, Yifu Guan

**Affiliations:** ^1^School of Intelligent Medicine, China Medical University, Shenyang, China; ^2^School of Nursing, China Medical University, Shenyang, China; ^3^Department of Biochemistry and Molecular Biology, China Medical University, Shenyang, China

**Keywords:** cognitive function, visuospatial dysfunction, spatial navigation, cognitive assessment, aging, dementia

## Abstract

Visuospatial function is a critical aspect of cognitive abilities, encompassing visual perception, attention, memory, and adaptive responses to spatial changes. This paper reviews studies on human visuospatial function, spatial navigation, and factors contributing to visuospatial impairments. After introducing fundamental concepts of visuospatial function and spatial navigation, classical methods for assessing visuospatial performance are summarized. By examining recent advances in spatial navigation studies, this paper discusses factors influencing spatial navigation capabilities and explores how spatial navigation paradigms can be used to investigate visuospatial cognitive impairments. Finally, current limitations in spatial navigation research are highlighted. Overall, the current research has not yet reached definitive conclusions regarding visuospatial aspects. However, this paper aims to enhance the understanding of visuospatial dysfunction and spatial navigation, providing valuable references for future research.

## 1 Background

Visuospatial function is an essential component of cognitive ability, which progressively declines with aging (Alexander et al., 2012; Dykiert et al., 2012; Gonzales et al., 2022; Schulz et al., 2022; Zhang et al., 2023). Additionally, various conditions, such as Alzheimer’s disease (AD), mild cognitive impairment (MCI), brain injury, and depression, further exacerbate cognitive impairments (Brett et al., 2022; Huang et al., 2023; Khan et al., 2023; Kriesche et al., 2023; Nouraeinejad, 2024; Wang et al., 2024; Alzheimer’s and Dementia, 2024). Declines in cognitive functioning severely impact patients’ and their families’ quality of life, imposing significant psychological stress and caregiving burdens. Therefore, interventions targeting cognitive decline are also crucial. Cognitive deficits can be improved through various intervention methods, such as digital rehabilitation and music therapy (Giannouli et al., 2024). Rapid global aging has now led to numerous societal challenges, notably including elderly individuals getting lost and experiencing difficulties in daily life due to impaired visuospatial abilities.

Observing behavioral performance during spatial navigation tasks across diverse populations holds substantial scientific and clinical value for investigating visuospatial functions. Human spatial navigation involves multiple cognitive processes—such as working memory, perception, and attention—which differ across age groups and clinical populations (Van Gerven et al., 2012; Mendez-Lopez et al., 2020; Coutrot et al., 2022).

This paper reviews the relationship between visuospatial function and aging, explores its association with various diseases, examines current clinical methods used for assessing visuospatial abilities, and discusses spatial navigation tasks as a promising approach for evaluating these cognitive functions. Additionally, it summarizes existing methodological limitations and aims to provide valuable guidance for future research on human visuospatial cognition.

## 2 Visuospatial function and spatial navigation

### 2.1 Concepts of visuospatial function and spatial navigation

Visuospatial function refers to as the brain’s ability to perceive and represent visual information from the surrounding environment, enabling the understanding and manipulation of spatial relationships. It encompasses visuospatial perception, working memory, attention, and executive functions (McGrew, 2009). Visuospatial perception involves the brain’s processing of spatial information through vision, utilizing complex neural networks to transform visual signals into three-dimensional representations of object positions (Kravitz et al., 2011). Visuospatial working memory pertains to the temporary storage, consolidation, and retrieval of visual-spatial information. Visuospatial attention involves searching for and identifying visual stimuli and their locations within the environment (Pal et al., 2016). Visuospatial executive ability supports higher cognitive processes such as arithmetic operations and financial decision-making, playing a crucial role in daily life and decision-making (Papp et al., 2011; Muffato et al., 2022; Giannouli and Tsolaki, 2023). These functions rely on the collaborative activity of multiple brain regions, particularly the posterior parietal cortex and visuomotor areas (Bai et al., 2021). The posterior parietal cortex plays a pivotal role in spatial attention and visuospatial information processing, including tracking moving targets, object localization in three-dimensional space, and integration of visual information (Burke et al., 2015). Visuomotor areas specialize in processing visual motion information, such as object orientation, motion velocity, and smooth pursuit of moving objects (Konen and Kastner, 2008).

Spatial navigation is a multifaceted behavior involving the integration of spatial information to accomplish environmental recognition, landmark identification, movement planning, and navigation (Garg et al., 2024). This process strongly integrates visual processing and cognitive abilities. Spatial navigation strategies are generally classified into two categories: egocentric navigation and allocentric navigation (Vijayabaskaran and Cheng, 2022). Egocentric navigation employs the navigator itself as the reference to determine the relative positions of surrounding objects. In contrast, allocentric navigation establishes an external coordinate system, calculating positions of the navigator, destinations, and landmarks to facilitate accurate path planning (Burgess, 2008).

### 2.2 Classical evaluation of visuospatial function

Currently, visuospatial functions are primarily evaluated using neuropsychological scale-based assessments and experimental paradigms. Commonly used cognitive assessment scales include the Mini-Mental State Examination (MMSE), Montreal Cognitive Assessment (MoCA), and MATRICS Consensus Cognitive Battery (MCCB). Each scale comprises tasks and questions covering multiple cognitive domains, including memory, language, visuospatial function, attention, and executive function, with clearly defined scoring criteria. For visuospatial assessment, the MMSE requires participants to draw two intersecting pentagons, while the MoCA asks participants to draw a cube. Both tests allocate a maximum of 3 out of 30 points specifically for visuospatial tasks. The MCCB employs a maze navigation task, in which participants must find their way from an entrance to an exit through trial-and-error exploration. Performance is scored based on maze complexity and completion time.

Another widely adopted category involves figure reproduction tasks, such as the Clock Drawing Test and the Rey–Osterrieth Complex Figure Test. The Clock Drawing Test requires participants to accurately draw a clock face with 12 correctly placed numbers and three appropriately positioned hands indicating a specified time. Participants’ drawings are rated from 1 to 4, depending on completeness and accuracy. The Rey–Osterrieth Complex Figure Test evaluates visuospatial memory by requiring participants to memorize and subsequently reproduce a complex geometric figure. Similar reproduction tasks are also integrated within the Visual Object and Space Perception Battery (VOSP) (Quental et al., 2013). Generally, neuropsychological scale-based tests are designed for rapid clinical screening to identify cognitive dysfunctions.

Additionally, several clinical assessments specifically target individual visuospatial functions. For instance, the Brief Visuospatial Memory Test (BVMT) primarily assesses visuospatial memory (Kane and Yochim, 2014); the Trail Making Test (TMT) evaluates visual scanning and processing speed (Park and Schott, 2022); and the Corsi Block-Tapping Task measures visuospatial working memory and spatial sequence recall (Huang et al., 2023).

These traditional tests exhibit certain limitations (Tragantzopoulou and Giannouli, 2024). A common characteristic of these assessments is that participants are required to interpret visual cues from scales or experimental objects and then perform simple tasks, including reproductions or conversions from textual to visual representations. The primary limitation of these conventional methods is their reliance on two-dimensional (2D) plane-based tasks, lacking translation processes from two-dimensional visual information into true three-dimensional (3D) spatial cognition. Consequently, these methods inadequately assess participants’ authentic 3D spatial perception, spatial orientation, and landmark recognition abilities. These assessment tools still face issues such as a lack of standardized criteria and insufficient detection capability for mild cognitive impairment (MCI) (Tragantzopoulou and Giannouli, 2024). Furthermore, scale-based assessments are susceptible to subjective bias, influenced by participant motivation and environmental interference during testing, thereby reducing the accuracy and validity of their outcomes.

## 3 Assessment of visuospatial function using spatial navigation

### 3.1 Age effects on spatial navigation function

Human spatial navigation ability depends on visuospatial function and is influenced by factors such as occupational experience, physical health, and aging. Functional and structural neuroimaging studies have demonstrated that the hippocampus is the primary brain region responsible for spatial navigation (Sosa and Giocomo, 2021), with the parahippocampal cortex, retrosplenial cortex, dorsal striatum, and posterior parietal cortex forming an extended neural network crucial to navigation (Baumann and Mattingley, 2021). With advancing age, the hippocampus and associated brain regions exhibit gradual volume reduction, resulting in notable differences in allocentric and egocentric navigation strategies between older and younger adults. Age-related impairments become particularly evident in tasks involving the recall of spatial landmarks and the recognition of environmental contexts. Older adults commonly experience difficulties in accurately processing positional, sequential, and directional landmark information during route learning. Wayfinding, specifically linked to hippocampal functions, and route learning, largely mediated by the caudate nucleus, both demonstrate age-related differences in spatial knowledge acquisition (Head and Isom, 2010). Moreover, Bécu et al. (2023) indicated that allocentric navigation strategies present greater challenges for older adults, whereas egocentric strategies remain relatively preserved with aging.

### 3.2 Effects of demographic and environmental factors on spatial navigation

Recent studies have demonstrated that demographic factors (e.g., gender, residential location) and environmental factors (e.g., living environment) significantly influence spatial navigation abilities. Males and females exhibit distinct preferences in selecting navigation strategies (Levine et al., 2016; Hegarty et al., 2023). Specifically, males tend to prefer egocentric, path-based navigation strategies, reflecting their greater confidence in accurately perceiving directional orientation and spatial positioning (Mendez-Lopez et al., 2020). In contrast, females typically favor allocentric, landmark-based strategies, relying more heavily on visual landmarks for orientation in familiar environments rather than their own relative spatial positioning (Van Gerven et al., 2012).

Residential location and environmental context also impact navigation strategies, spatial cognition and route learning. The cultural and geographical attributes of an individual’s living environment significantly affect cognitive processes and mental health (Van Praag et al., 2000). Individuals tend to perform better when navigating environments with topological structures similar to those they experienced during childhood (Coutrot et al., 2022). Urban environments typically feature complex layouts with abundant visual landmarks, leading residents to rely primarily on landmark-dependent navigation strategies. In contrast, rural settings usually lack distinct visual markers and are characterized by natural geographical features such as mountains and rivers. Consequently, rural inhabitants tend to utilize global spatial perception and directional orientation rather than relying on specific visual landmarks (Weisberg et al., 2014; Ekstrom and Isham, 2017; Spiers et al., 2023). Additionally, unique environmental contexts, such as deserts with sparse landmarks and monotonous visual features, can foster superior directional orientation and path inference skills among local residents (Foo et al., 2005).

Other factors, including educational background, occupational characteristics, cultural differences, and individual variability, further influence spatial navigation performance. Therefore, it is crucial to control for these potential confounding factors in experimental design to minimize biases and ensure accurate and reliable research findings.

### 3.3 Spatial navigation experiments for assessing visuospatial function

#### 3.3.1 Spatial navigation experiments in real-world environments

Gazova et al. (2013) designed a real-world spatial navigation experiment to investigate navigation abilities across different age groups. The experiment consisted of three distinct tasks: allocentric-egocentric, egocentric, and allocentric navigation. Participants navigated real-world environments on foot, while their movement trajectories were recorded. The primary evaluation metric was the average distance error between participants’ actual paths and target locations. Results indicated that cognitively healthy older adults exhibited notable impairments in allocentric navigation, while their egocentric navigation and spatial learning abilities were partially preserved. Real-world visuospatial assessments such as this offer high ecological validity, closely resembling everyday navigation scenarios. However, the method requires specialized testing facilities and equipment, limiting its scalability. Additionally, participation is restricted to individuals with sufficient independent mobility.

#### 3.3.2 Virtual spatial navigation experiment based on a Y-maze

**FIGURE 1:**
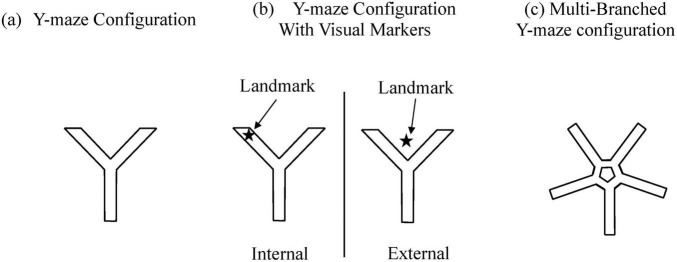
Several different Y-maze construction paradigms.

The rapid advancement of computer technology has provided efficient and practical tools for designing virtual environments in spatial navigation experiments. These experimental paradigms originated from the Morris water maze, a classic behavioral task developed in the 1980s (Othman et al., 2022) (Figure 1).

Ramanoël et al. (2022) developed a 3D virtual Y-maze, placing participants within the maze to locate specific targets using visual cues. Researchers evaluated participants’ navigation performance based on accuracy rate and completion time, revealing that manipulating visual landmarks could stabilize or enhance performance. Further analyses indicated a stronger reliance on egocentric navigation strategies among older adults. Bécu et al. (2023) refined this paradigm by positioning distinct visual landmarks prominently in mid-air outside the virtual Y-maze. Participants began at one end of the maze, choosing a pathway at a central junction; successful completion required selecting the correct endpoint. Metrics analyzed included attention allocation, memory retention, and spatial reasoning. Results indicated that adolescents and older adults frequently failed under landmark-rich conditions, while young adults generally succeeded. The authors hypothesized that adolescents and older adults predominantly employed egocentric strategies, whereas young adults utilized allocentric navigation strategies.

Bellassen et al. (2012) expanded the virtual Y-maze by adding three additional branches, resulting in a central pentagonal ring connected to five radially symmetrical paths. The experiment involved two tasks: a temporal memory test, requiring participants to recall the sequential routes from a previously learned path, and a spatial memory test, which involved recalling environmental cues and identifying correct locations on a map. Researchers evaluated visuospatial abilities by examining working memory, visuoconstructional skills, and executive function. Experimental outcomes indicated spatial and temporal memory deficits in AD patients. Importantly, the temporal memory test revealed no significant age-related impairment and successfully differentiated participants with AD and amnestic mild cognitive impairment (aMCI) from age-matched healthy controls, demonstrating high sensitivity and specificity.

#### 3.3.3 Virtual spatial navigation experiments simulating real-world scenarios

**FIGURE 2:**
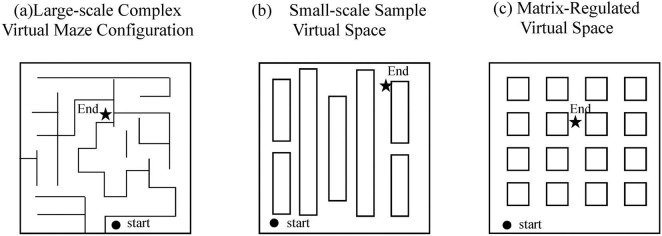
Several different paradigms for constructing virtual spaces.

The virtual spatial navigation experiments simulating real-world scenarios typically involve environments featuring grid-like streets, neighborhoods and diverse buildings. This realistic design enhances participants’ sense of immersion, thereby increasing experimental validity and acceptability (Figure 2).

Morganti et al. (2013) developed a virtual reality maze test (VRMT) comprising two tasks: sequential navigation and route tracing. In the former task, participants were required to plan a path from the maze entrance to the exit. The latter task asked participants to recall correct turning directions at intersections, guided by a virtual reality roadmap (VR-RMT). Performance was evaluated using navigation accuracy rates and the accuracy of turn-direction recall. Results indicated that the AD group performed worse than the control group, demonstrating lower navigation accuracy and fewer successful completions. This suggests that spatial navigation assessments might facilitate early screening for Alzheimer’s disease.

Tu et al. (2017) designed a virtual supermarket environment, incorporating counters displaying differently colored products as visual landmarks. Participants navigated to designated locations based on experimental instructions. Performance metrics included accuracy rates and deviation values from correct target locations. The AD group exhibited significantly poorer performance in both egocentric and allocentric navigation compared to controls. Notably, participants with the behavioral variant of frontotemporal dementia demonstrated similarly impaired navigation capabilities.

Kimura et al. (2019) utilized virtual reality technology to construct a grid-like virtual space with distinctive landmarks placed throughout the environment. By varying grid dimensions, landmark appearances, starting positions and target locations, the study assessed participants’ navigation proficiency in reaching designated target rooms. The elderly group consistently showed lower accuracy rates across all experimental conditions compared to younger participants, with the most pronounced deficits observed when landmarks were displaced or when starting locations changed. Researchers suggested younger adults subconsciously utilized landmarks more effectively, demonstrating greater adaptability to environmental variations.

Van Der Ham et al. (2020) adopted a distinct methodological approach by designing a passive observational spatial task. Unlike previous studies requiring active navigation, participants passively viewed a three-dimensional environmental video and subsequently completed questionnaires assessing their recall of spatial cues and orientations. Results indicated an age-related linear decline in males’ advantage in landmark knowledge, with individual variability observed. The authors proposed that this passive observational paradigm could serve as a clinical tool for identifying early-stage visuospatial dysfunction.

#### 3.3.4 Spatial navigation experiments embedded in video games

In recent years, spatial navigation experiments utilizing video games have emerged as an engaging alternative to traditional tasks, significantly improving participant compliance and task enjoyment. Coutrot et al. (2022) embedded spatial navigation tasks within the video game “Sea Hero Quest”, in which participants navigated from designated starting points to checkpoints following specific routes. Navigation difficulty progressively increased with advancing game levels. The researchers observed that participants navigated more effectively in environments resembling those experienced during their upbringing, highlighting stable associations between developmental environments and cognitive function across the lifespan.

Merriman et al. (2022) designed another spatial navigation experiment using the video game “CityQuest”. Participants first memorized the spatial locations of four targets, then navigated to these targets while selecting optimal routes and avoiding obstacles. Performance was evaluated by analyzing metrics such as decision-making time and navigation efficiency. Results indicated that spatial navigation training improved older adults’ utilization of egocentric strategies, particularly demonstrating enhanced navigation abilities in obstacle-rich virtual environments.

## 4 Research findings on spatial navigation and visuospatial function

### 4.1 Effects of aging factors

Human exhibits significant age-related differences in visuospatial information processing and spatial navigation tasks (Barnes, 1979; Othman et al., 2022). Muffato et al. (2020) demonstrated an age-associated decline in visuospatial function, consistent with findings from other similar experimental paradigms (Alexander et al., 2012; Dykiert et al., 2012; Schulz et al., 2022; Zhang et al., 2023).

Human visual working memory capacity declines with age (Liu M. et al., 2021). This deterioration correlates strongly with reduced efficiency in processing complex visual information and integrating visual features, suggesting that older adults experience notable impairments in evaluating spatial attributes such as distances and viewing angles (Brockmole and Logie, 2013). In complex real-world visual scenarios, older adults often require extended time for spatial information processing (Meng et al., 2019). Additionally, age-related decreases in attentional resources and efficiency of visual processing prolong response times in older adults during visual search tasks (Madden and Langley, 2003; Ebaid and Crewther, 2019). Finally, given that visuospatial functions depend on coordinated activity across multiple brain regions, age-related degeneration in these areas further contributes to visuospatial impairments (Almanza-Sepúlveda et al., 2018; Yoo et al., 2019; Bai et al., 2021; Abdolalizadeh et al., 2022). It should be kept in mind that pathological changes such as glaucoma associated with normal aging can also exert direct impact visuospatial functions by affecting spatial contrast sensitivity, motion perception, and visual processing speed (Owsley, 2011).

Older and younger adults also differ in their preferred spatial navigation strategies. Specifically, older adults tend to rely more heavily on egocentric navigation, whereas younger adults predominantly utilize allocentric strategies (Bécu et al., 2023). The preference for egocentric strategies among older adults is attributed to age-related functional declines in allocentric processing, closely linked to hippocampal dysfunction and structural deterioration in the prefrontal and parahippocampal regions (Meulenbroek et al., 2004; Antonova et al., 2009). Previous studies have demonstrated that allocentric spatial memory critically depends on hippocampal integrity (Holdstock et al., 2000; Ramos and Morón, 2022), whereas egocentric memory primarily engages the caudate nucleus (Iaria et al., 2003; Vijayabaskaran and Cheng, 2022). Although the caudate nucleus also undergoes age-related atrophy (Weerasekera et al., 2023), deficits in allocentric navigation strategies are generally more pronounced than those in egocentric navigation (Harris et al., 2012). These physiological changes associated with brain aging likely underlie the increased reliance on egocentric strategies observed in older adults.

### 4.2 Effects of cognitive disease factors

The neural mechanisms underlying visuospatial function involve coordinated activity across multiple brain regions. Pathological brain changes caused by neurodegenerative diseases inevitably impair visuospatial abilities. Declines in visuospatial functions have been reported even during the early stages of AD, including MCI (Hort et al., 2007; Ciafone et al., 2022; Derbie et al., 2022). Consequently, visuospatial assessments hold promise for early screening of both MCI and AD (Chapman et al., 2011). Neurodegenerative diseases significantly impair various visuospatial abilities, including spatial memory, attention, perception, and decision-making. Spatial navigation ability, thus, could be a predictive indicator for predementia syndromes (e.g., MCI) in older adults, and impairments in spatial navigation are also an early feature of AD (Verghese et al., 2017). Researchers have identified visuospatial working memory deficits as notable prodromal symptoms in patients with AD (Schmid et al., 2013). Further studies have demonstrated that individuals with AD exhibit significant difficulties forming and recalling cognitive maps due to impaired hippocampal functioning (Coughlan et al., 2018). The hippocampus plays a critical role in differentiating environmental from spatial configurations (Maurer and Nadel, 2021) and encodes spatial-environmental features into cognitive maps (Knudsen and Wallis, 2021). In AD, hippocampal degeneration directly compromises these functions (Ekstrom et al., 2003). Additionally, cognitive impairment significantly disrupts patients’ spatial memory, rendering them unable to accurately retrieve routes or landmark information (Coughlan et al., 2018). Attentional resource allocation capacity is also impaired, resulting in difficulties filtering irrelevant information and accurately identifying navigational cues (Liao et al., 2024). Furthermore, AD and other neurodegenerative disorders can lead to dysfunction in the parietal lobe, adversely affecting visual information processing and interpretation (Liu N. et al., 2021). Consequently, patients with AD and MCI exhibit deficits in route learning and orientation, which severely impair their directional judgment and spatial orientation (deIpolyi et al., 2007; Yew et al., 2012). As evidenced here, the neural basis of visuospatial functions manifested in spatial navigation tasks is distinct from other cognitive functions, and their decline reflects functional deterioration in specific brain regions rather than a generalized decline in overall cognitive resources.

Cognitive dysfunction also distinctly affects the two primary spatial navigation strategies (allocentric and egocentric). When only visual cues were available, AD patients showed markedly impaired navigation performance, reflecting compromised allocentric navigation strategies (Kalová et al., 2005; Morganti et al., 2013). These allocentric impairments are closely related to pathological changes in the hippocampus. Additionally, AD patients experience difficulties in judging egocentric aspects such as their starting positions and estimating distances (Tu et al., 2017), essential for egocentric navigation. Moussavi et al. (2022) experimentally confirmed impairments in egocentric navigation in AD patients. Similarly, MCI patients exhibit deficits in both egocentric and allocentric memory (Tuena et al., 2021). Compared with age-matched healthy controls, MCI patients display poorer navigation performance in both strategies, though their deficits are less severe than those observed in AD patients (deIpolyi et al., 2007; Hort et al., 2007; Laczó et al., 2011; Tuena et al., 2021). Furthermore, impairments differ among MCI subtypes: patients with amnestic multidomain MCI show deficits in both allocentric and egocentric navigation (Hort et al., 2007), whereas amnestic single-domain MCI patients exhibit impairments only in allocentric navigation. Non-amnestic MCI patients typically perform similarly to healthy controls (Lithfous et al., 2013). Importantly, allocentric navigation tasks can effectively distinguish between MCI patients with hippocampal-related memory deficits and those with retrieval impairments caused by frontal cortical damage (Laczó et al., 2011). Although many researches have referred these two navigation strategies, questions remain unresolved. Is navigation truly limited to egocentric and allocentric types? Do navigators exclusively rely on a single strategy during real-world navigation? How do these two strategies switch from one to another to adapt the environmental changes? Definitive answers are urgently needed for these issues.

### 4.3 Limitations in current visuospatial function research

Current research on spatial navigation primarily emphasizes navigational performance itself, with limited attention given to the relationship between navigation and visuospatial abilities. Visuospatial impairments may disrupt the accurate processing of spatial cues, consequently impairing navigation performance. However, few experimental designs explicitly control for visuospatial factors as potential confounders. Moreover, although numerous studies focus on navigation strategies, some infer strategy selection merely based on the presence of landmarks, neglecting scenarios in which landmarks are available but not consciously utilized by participants. This methodological oversight could introduce unintended biases. Consequently, there is a lack of objective criteria to accurately identify participants’ chosen navigation strategies.

Furthermore, critical cognitive processes underlying navigational decisions remain insufficiently explored, such as participants’ reasoning behind incorrect route selections, their attentional allocation at decision points (e.g., intersections), and their conscious awareness of spatial cues. Many existing studies focus on quantifying navigation errors to highlight age-related differences and subsequently utilize neuroimaging techniques like functional MRI to determine whether such errors result from normal aging or pathological conditions. However, real-world navigation frequently involves self-correction behaviors—individuals often recognize errors shortly after they occur, return to the error point, reorient themselves, and successfully adjust their routes. Remarkably, very few studies have examined these adaptive self-correction processes. Ignoring such behaviors significantly limits the ecological validity and practical value of spatial navigation research, as self-correction mechanisms might closely reflect underlying physiological and cognitive changes associated with aging and cognitive decline. In addition, the application of virtual reality technology in assessing visuospatial functions shows promising potential, but it requires overcoming adaptability challenges faced by elderly individuals with such complex technologies (Tragantzopoulou and Giannouli, 2024).
